# Anti-polymyositis/scleroderma antibody-positive myositis exhibits greater cutaneous T cell activation than anti-ARS antibody-positive myositis

**DOI:** 10.1016/j.jdin.2025.12.014

**Published:** 2026-01-22

**Authors:** Norika Akashi, Mariko Ogawa-Momohara, Yoshinao Muro, Takashi Yokoyama, Satoshi Kamiya, Yuta Yamashita, Haruka Koizumi, Takuya Takeichi, Masashi Akiyama

**Affiliations:** Department of Dermatology, Nagoya University Graduate School of Medicine, Nagoya, Aichi, Japan

**Keywords:** anti-ARS antibody, anti-PM/Scl antibody, dermatomyositis, skin immunopathology, T cell exhaustion

*To the Editor:* Anti-polymyositis/scleroderma (PM/Scl) antibodies were originally reported in patients with overlapping myositis and systemic sclerosis and are detected in approximately 5% to 8% of myositis cases, indicating a relatively low frequency. Recent analyses have shown that anti-PM/Scl-positive myositis shares several clinical features with aminoacyl-tRNA synthetase (ARS) antibody-positive myositis, including chronic interstitial lung disease and mechanic’s hands,^1^ with the latter occurring more frequently in patients with anti-PM/Scl antibodies. We previously reported that marked hyperkeratosis extending to the palms and soles is a characteristic finding in anti-PM/Scl-positive myositis^2^ (Supplementary Fig 1, available via Mendeley at https://data.mendeley.com/datasets/z66tb7xckg/1). Given the similar cutaneous manifestations seen in both anti-PM/Scl- and anti-ARS-positive myositis, we aimed to clarify how the mechanisms underlying keratinization differ between these 2 groups. We conducted a retrospective case–control study using RNA sequencing and immunofluorescence staining of formalin-fixed, paraffin-embedded skin biopsies obtained from hyperkeratotic sites of 10 Japanese patients with idiopathic inflammatory myopathy (6 anti-ARS positive and 4 anti-PM/Scl positive) and 5 healthy controls (Supplementary Tables I and II, available via Mendeley at https://data.mendeley.com/datasets/z66tb7xckg/1). From the differentially expressed protein-coding genes between anti-PM/Scl- and anti-ARS-positive myositis, we extracted immune- and epithelial-related genes and visualized 47 significant differentially expressed protein-coding genes through heatmaps and volcano plots. Unexpectedly, epithelial-related genes were more highly expressed in anti-ARS-positive myositis, whereas immune-related genes were predominantly upregulated in anti-PM/Scl-positive myositis (Supplementary Fig 2, available via Mendeley at https://data.mendeley.com/datasets/z66tb7xckg/1, Fig 1).

**Fig 1:**
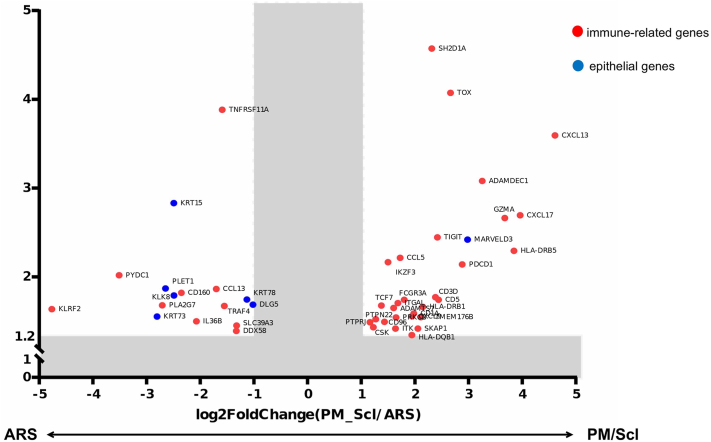
Differences in gene expression in lesional skin between anti-PM/Scl antibody-positive myositis and anti-ARS antibody-positive myositis. Gene expression profiling reveals that among the most differentially expressed genes between the anti-PM/Scl antibody-positive myositis patients (*n* = 4) and the anti-ARS antibody-positive myositis patients (*n* = 6), there are more immune-related genes in anti-PM/Scl antibody-positive myositis and more epithelial genes in anti-ARS antibody-positive myositis than in anti-PM/Scl antibody-positive myositis. Immune-related genes are shown in *red* and epithelial genes in *blue*. *ARS*, Aminoacyl-tRNA synthetase.

Half of the top 20 differentially expressed protein-coding genes in anti-PM/Scl-positive myositis were T cell-associated genes (Supplementary Table III, available via Mendeley at https://data.mendeley.com/datasets/z66tb7xckg/1). Comparisons of the expression levels of these T cell-related RNAs across the 3 groups (anti-PM/Scl-positive myositis, anti-ARS-positive myositis, healthy controls) showed the anti-PM/Scl group to have significantly higher expression than the other 2 groups (Fig 2). Genes associated with antigen presentation and TCR activation were also significantly increased (Supplementary Fig 3, available via Mendeley at https://data.mendeley.com/datasets/z66tb7xckg/1). CD3 immunofluorescence staining revealed a significant increase in T cell infiltration in anti-PM/Scl-positive myositis compared with healthy controls, whereas this was not observed in anti-ARS-positive patients, further supporting the idea of T cell enrichment in anti-PM/Scl-positive myositis (Supplementary Fig 4, available via Mendeley at https://data.mendeley.com/datasets/z66tb7xckg/1). Notably, the exhausted T cell markers TOX, PDCD1, and TIGIT were significantly upregulated in anti-PM/Scl-positive patients. Exhausted T cells arise when chronically stimulated T cells exposed to persistent inflammation or self-antigens fail to receive sufficient costimulatory signals. In autoimmune diseases such as rheumatoid arthritis, ANCA-associated vasculitis, systemic lupus erythematosus, and primary Sjögren’s syndrome, increased populations of exhausted T cells have been associated with fewer relapses and a more favorable disease course.^3^ Thus, the enrichment of exhausted T cells may help to explain why interstitial lung disease in anti-PM/Scl-positive myositis tends to respond well to immunosuppressive therapy.^1^ Although the anti-PM/Scl group displayed pronounced hyperkeratosis clinically, our transcriptomic analysis had an unexpected finding: a predominance of T cell-related—rather than keratinocyte-related—genes. Cytotoxic T cell-derived GZMA^4^ and keratinocyte-derived chemokines involved in leukocyte recruitment, CXCL9 and CCL5,^5^ were elevated in anti-PM/Scl-positive myositis. These findings raise the possibility of a pathogenic loop between activated T cells and keratinocytes: T cell-derived cytotoxic mediators and keratinocyte-derived chemokines may mutually amplify inflammation, contributing to the marked hyperkeratosis observed in anti-PM/Scl-positive myositis (Supplementary Fig 5, available via Mendeley at https://data.mendeley.com/datasets/z66tb7xckg/1).(495/500).

**Fig 2:**
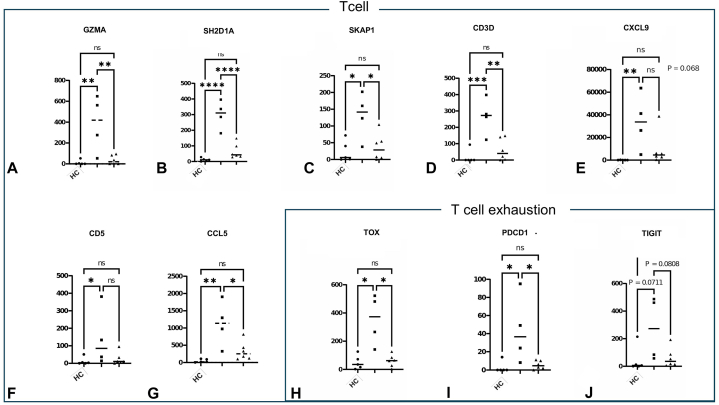
Gene expression analysis of T cell-related genes in anti-PM/Scl antibody-positive myositis and anti-ARS antibody-positive myositis skin lesions. **A-G,** Expression levels of T cell genes (*GZMA, SH2D1A, SKAP1, CD3D, CXCL9, CD5*) for the 3 clinical groups. **H-J,** Expression levels of T cell exhaustion markers (*TOX, PDCD1, TIGIT*) for the 3 clinical groups. ∗ *P* < .05, ∗∗ *P* < .01, ∗∗ *P* < .001, ∗∗∗∗ *P* < .0001; *HC*, Healthy control; *ARS*, aminoacyl-tRNA synthetase.

## Conflicts of interest

None disclosed.
